# Dental Restorative Materials for Elderly Populations

**DOI:** 10.3390/polym13050828

**Published:** 2021-03-08

**Authors:** Yuyao Huang, Bingqing Song, Xuedong Zhou, Hui Chen, Haohao Wang, Lei Cheng

**Affiliations:** 1 State Key Laboratory of Oral Diseases, West China School of Stomatology, National Clinical Research Center for Oral Diseases, Sichuan University, Chengdu 610064, China; huangyuyaott@163.com (Y.H.); sbqelaine@163.com (B.S.); zhouxd@scu.edu.cn (X.Z.); 2 Department of Cariology and Endodontics, West China School of Stomatology, Sichuan University, Chengdu 610041, China; 3 Department of Operative Dentistry and Endodontics, Guanghua School of Stomatology, Guangdong Provincial Key Laboratory of Stomatology, Sun Yat-sen University, Guangzhou 510055, China; chenhuidentist@163.com

**Keywords:** elderly populations, commercial dental restorative materials, composite materials, polymer and biopolymer, antimicrobial materials, remineralization materials, self-healing materials

## Abstract

The incidence of dental caries, especially root caries, has risen in elderly populations in recent years. Specialized restorative materials are needed due to the specific site of root caries and the age-related changes in general and oral health in the elderly. Unfortunately, the restorative materials commonly used clinically cannot fully meet the requirements in this population. Specifically, the antibacterial, adhesive, remineralization, mechanical, and anti-aging properties of the materials need to be significantly improved for dental caries in the elderly. This review mainly discusses the strengths and weaknesses of currently available materials, including amalgam, glass ionomer cement, and light-cured composite resin, for root caries. It also reviews the studies on novel anti-caries materials divided into three groups, antimicrobial, remineralization, and self-healing materials, and explores their potential in the clinical use for caries in the elderly. Therefore, specific restorative materials for caries in the elderly, especially for root caries, need to be further developed and applied in clinical practice.

## 1. Introduction

The aging of the population is a worldwide phenomenon, and increased life expectancy impacts the human oral health status. The elderly face challenges of age-related changes in general and oral health. The incidence of dental caries is high in elderly populations [1], which is a growing public health issue due to the dramatic increases in tooth retention rate. According to the Fourth Oral Epidemiological Survey of China in 2015, the prevalence of dental caries was 98.0% in people aged 65 to 74, and the elderly were more likely to develop root caries than coronal caries. The prevalence of root caries in this group was 61.9%, only 3% of which were restored. In the USA, it was estimated that 60% of people > 65 years old suffered from root caries [2]. Similar to other caries, root caries is mainly treated by a restorative procedure, usually ending up in failure due to the high proportion of secondary caries and restoration fracture [3]. Caries in the elderly, leading to a higher prevalence of recurrent caries, require more oral treatments than other caries, with a heavy economic burden on society and the medical health service. Specific oral microorganisms [4] and changing anatomic structures require specialized restorative materials to treat caries in the elderly. However, the clinically available restorative materials, including amalgam, glass ionomer cement, and light-cured composite resin, cannot fully meet the requirements. Therefore, the antibacterial, adhesive, remineralization, mechanical, and anti-aging properties of the materials should be dramatically enhanced to meet the restorative needs of the elderly. This review was undertaken to discuss the strengths and weaknesses of the currently available materials and review previous studies on novel anticariogenic materials that can be potentially used in the clinic to restore caries in the elderly.

## 2. The Challenges of Dental Restorative Materials in the Elderly

With the impaired immune function in the elderly, the local oral environment faces many challenges concerning the restorative treatment of dental caries.

**Figure 1:**
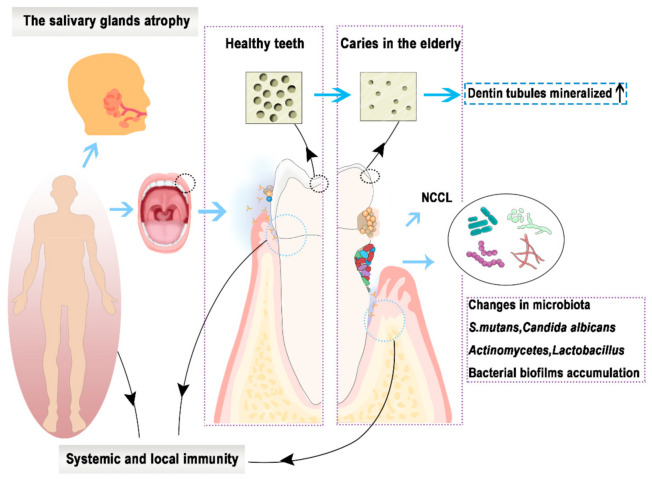
The elderly are confronted with the following changes: Salivary gland atrophy, susceptibility to periodontitis, non-carious cervical lesions (NCCL), and root caries.

Figure 1 summarizes the challenges of dental restorative materials in elderly populations.

Restorative materials for dental caries in the elderly should exhibit antibacterial properties. The decreased salivary flow (i.e., hyposalivation) and reduced bicarbonate concentration in the elderly lead to a weak acidic local oral environment [5,6]. The cementum and root dentin are more vulnerable to acid attack and demineralization in a weak acidic environment, resulting in root caries. In the root caries, in addition to Streptococcus mutans [7] and Lactobacillus acidophilus, Actinomyces species and Candida albicans are also detected in large amounts, which might play critical roles in the development of these lesions [8,9]. Therefore, the local environment of root caries is more diverse and the treatment of root caries in the elderly needs to target a variety of microorganisms. In the elderly, wearing removable partial dentures, frequent sugar intake, poor oral hygiene [10], and rougher surface of teeth root would increase bacterial biofilms accumulation. Moreover, the elderly are more susceptible to microbial infections as their saliva clearance rate and immunity are compromised. Changes in microbial flora, increased biofilms adhesion, decreased saliva clearance, and immunity give rise to a higher demand for the antibacterial property of dental materials in the elderly. The property includes inhibiting the growth of *Streptococcus mutans* [11], *Lactobacillus acidophilus*, *Actinomyces species*, and *Candida albicans* [12], reducing the adhesion of bacterial biofilms, and being biologically safe.

Restorative materials in the elderly should also provide remineralization and adhesive properties. The elderly are more susceptible to gingival recession and periodontitis [13], leading to the cementum and dentin of root surfaces to be exposed. In addition, a thin cementum can be lost by brushing teeth or dental plaque biofilm acid, exposing the root dentin. The carbonate content of hydroxyapatite (HA) in dentin increases with age, resulting in increased dentin sensitivity to acid and making it more soluble than other ages [14]. Therefore, exposed root surfaces with thin or even no cementum are vulnerable to acid attack, resulting in root caries. In other words, the elderly are more vulnerable to demineralization. As a result, dentin remineralization is necessary for root caries. For the elderly, ideal remineralized restorative materials should be able to carry out the following: Remineralize the demineralized dentin, have a long-term remineralization effect, be suitable for patients with a dry mouth (i.e., xerostomia), and not promote calculus production.

On the other hand, it is difficult to isolate the moisture in the periodontal pockets when root caries is close to gingival during restorative treatments [15]. It is worth noting that root caries is usually dentin caries, challenging dentists in two ways. One is resin-dentin bonding. The “hybrid layer” formed by the resin-dentin diffusion has a great influence on the bonding strength of the restorative materials [16]. However, the dentin tubules in the elderly are degenerative and mineralized [17]. Matrix metalloproteinases (MMPs) from dentin, activated by acid, can degrade the hybrid layer [18]. Therefore, the mineralized dentin of the elderly cannot be etched well, resulting in a weak adhesion layer. Hence, adhesion to dentin might be achieved by self-etching adhesives according to the adhesion-decalcification concept. In general, the periodontal condition and dentinal structure necessitate that the dental restorative materials in the elderly must promote dentin remineralization and achieve strong bonding with the root surface.

Other challenges for dental restorative materials in the elderly are mechanical and anti-aging properties. Apart from the hardness and elastic modulus, the fracture toughness of dentin reduced with the increasing age, making teeth more likely to be fatigued [19]. The restored teeth are also less resistant to fatigue cracks [20]. A study showed that the cervical area of the teeth is a stress concentration area during mastication [21,22]. As a result, root caries in the elderly is a high risk of tooth fracture. The chemical and physical factors in the oral environment such as microbial metabolites, saliva, gingival crevicular fluid, intraoral loads (forces) will degrade restorative materials. Over time, it will cause the occurrence of microleakage, change color of restorative materials, and increase roughness, eventually leading to secondary caries [23]. Therefore, dental restorative materials for the elderly require upstanding mechanical properties and should resist degradation due to the complex oral environment. First of all, the materials must be wear-resistant and absorb the occlusal force, with less stress shrinkage. They should also be able to repair cracks in the dental tissue, reducing the risk of secondary caries and tooth or restoration fracture.

Considering the physical and local physiological changes in the elderly, dental caries in the elderly should be treated with special care. The restorative materials for caries should have considerable antibacterial, remineralization, adhesive, mechanical, and anti-aging properties.

## 3. Currently Available Restorative Materials for the Elderly

### 3.1. Amalgam

Amalgam can release silver ions and mercury ions to inhibit the growth of *Streptococcus mutans* and *Actinomycete* biofilms, with no bacterial resistance [24]. It seldom causes microleakage and secondary caries, therefore, amalgam is suitable for subgingival cavities to a certain extent. However, to obtain good retention, the dental tissue has to be extensively removed, as the amalgam and the tooth can only be mechanically interlocked [25]. As a result, the remaining insufficient tissue on roots might not be able to bear too much occlusal force and is easily broken. However, this might be compensated by its elastic modulus, which is similar to dentin and its excellent mechanical properties [26]. In addition, amalgam can endure saliva and gingival crevicular fluid and is not degraded. Therefore, amalgam can be preserved in the oral environment for a long time compared to other restorative materials [27]. However, the disadvantages of amalgam are also evident. The leakage of mercury is a potential risk to human health [28], and the release of mercury into the environment causes environmental pollution. Moreover, the color of amalgam is too different from that of teeth, and it cannot meet the aesthetic needs of anterior root caries [29]. In summary, the disadvantages of amalgam outweigh its advantages. Amalgam is gradually being eliminated from clinical practice since other more suitable materials are widely used.

### 3.2. Glass Ionomer Cement

The glass ionomer cement (GIC) commonly used in clinical practice can be divided into three types: Conventional, resin-modified, and high-viscosity GIC [30]. Glass ionomer can release fluoride ions [31], therefore, it exhibits antibacterial and remineralization properties [32]. Studies have shown that glass ionomer can inhibit the growth of *Streptococcus mutans* and *Lactobacillus*, therefore, it can prevent secondary caries to a certain extent but cannot inhibit the growth of *Candida albicans* [33], one of the pathogenic bacteria responsible for root caries [34]. Since the release of fluoride ions is related to the glass ionomer composition, most high-viscosity and resin-modified glass ionomers have a lower total fluoride ion release than the conventional ones [35], therefore, conventional glass ionomers have better antibacterial properties [36]. The remineralization properties of glass ionomer are also related to the release of fluoride ions [37]. Glass ionomer can promote the remineralization of partially demineralized tooth hard tissues but cannot remineralize completely demineralized tooth hard tissues [38]. Concerning caries in the elderly, the existing glass ionomer has certain antibacterial and remineralization properties [38]. However, due to fact that the release of fluoride ions from glass ionomer is explosive in the early stages, decreasing slowly to a very low concentration in later stages, it does not have controlled release and long-term release [39], necessitating an improvement in this respect.

Glass ionomer has self-adhesive properties, through a chemical combination with the tooth hard structure, and can be used in a humid environment [40]. Except for the resin-modified glass ionomer, which requires light curing, other glass ionomer types do not require light curing [41], therefore, it is low-technique-sensitive, and the operating time is short. The treatment of root caries in the elderly might be hampered by gingival crevicular fluid exudation, making it difficult to prevent moisture [15]. In addition, the elderly cannot easily tolerate lengthy clinical procedures. Therefore, the self-adhesive properties of glass ionomer are advantageous in the treatment of caries in the elderly. The mechanical properties of conventional glass ionomers are the worst among all [42]. The hardness and long-term wear of modified high-viscosity glass ionomer and resin-modified glass ionomer are far lower than the conventional ones [43]. Since caries in the elderly, especially root caries, do not withstand high occlusal loads, the mechanical properties of glass ionomer are suitable for the elderly. In summary, conventional glass ionomers have good antibacterial and remineralization properties, and modified glass ionomers have better mechanical properties and retention rates. For caries in the elderly, glass ionomer is a good choice. However, further improvements are still necessary.

### 3.3. Light-Cured Composite Resin

Light-cured composite resin is more commonly used for the restorative treatments of dental caries in seniors. Composite resin restorations lack antibacterial properties and accumulate dental plaque [44]. It was reported that the percentage of *Streptococcus mutans* and *Candida albicans* in the dental plaque on the resin was higher than that of amalgam and GIC [45]. Biofilms might cause secondary caries [46] and materials aging. There are already some composite resins with fluoride ion-releasing ability for clinical applications, such as Beautifil II [47,48], Dyract Extra, etc. These fluorine-releasing composite resins can slowly release fluoride ions over a long time [49], considered to be beneficial for antimicrobial properties and remineralization. Conventional composite resins and tooth structures are bonded through adhesives. Self-etching adhesives are more suitable for dentin caries [50,51], but still difficult for the sclerotic aging dentin.

The mechanical properties of the composite resin are acceptable and better than the glass ionomer cement [52,53]. However, the particulate filler resin composite is of low mechanical strength, which may cause high local stress concentrations and damage the restoration. Fiber-reinforced composites (FRC), widely used in engineering, have been clinically approved for restorative dentistry for decades [54]. The physical and chemical properties and various research models of this engineering material have been widely studied in other fields [55]. Fibers in the FRC increase toughness and other physical properties of the material compared to the regular composite resin [56]. Fibers will prevent crack propagation through restorations and mimic the stress absorbing properties of dentin [57]. As dentin replacement, short fiber-reinforced composites, such as everX Flow and everX Posterior, display an exceptionally high fracture toughness and low wear depth [58]. Teeth filled by the FRC might be less likely to develop secondary caries and bulk fracture. However, a significant disadvantage of composite resin is the polymerization shrinkage, causing microleakage and affecting the bonding [59,60]. The mechanical properties of composite resins are often affected by aging and the degree of polymerization.

In conclusion, composite resins also have some advantages, such as good appearance, compressive strength [61], and wear resistance (Table 1). However, decreased mechanical properties, restoration fracture, pulp irritation [62], and secondary caries are common problems of composite resin restorations [63]. It is still urgent to improve the properties of light-cured composite resin to meet the requirements of restorative treatments of caries in the elderly.

## 4. Novel Anticariogenic Restorative Materials

**Figure 2:**
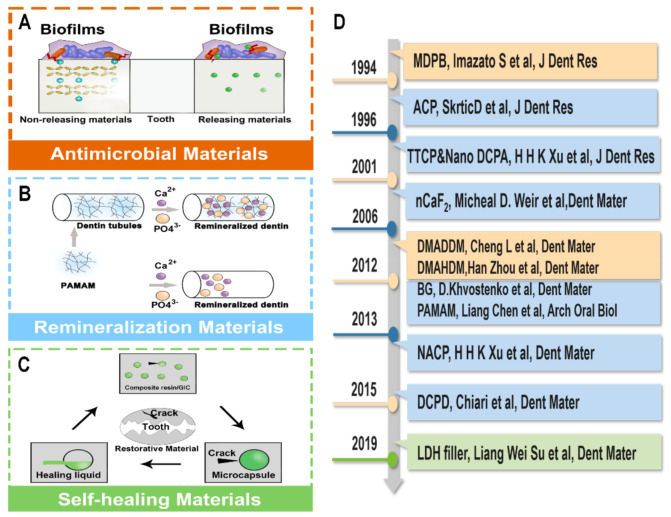
(**A**) Antibacterial materials. (**B**) Remineralization materials. (**C**) Self-healing materials. (**D**) Timeline.

Figure 2 summarizes the timeline of novel anticariogenic restorative materials.

### 4.1. Antimicrobial Materials

As mentioned previously, with changes in the types of cariogenic microorganisms, increased biofilms adhesion and secondary caries [64] in the elderly populations, dental restorative materials need to inhibit root caries biofilms and reduce the proportion of acid-producing bacteria [65,66]. However, restorative materials used clinically do not have ideal antimicrobial properties for the elderly. In recent years, some researchers have studied antimicrobial materials [67,68,69,70] that might be used for caries restorative procedures in the elderly. Those novel materials can reduce the number of cariogenic bacteria, have long-term antibacterial effects, and are expected to acquire both antibacterial and anti-aging properties. Antibacterial materials could be divided into non-releasing materials and releasing materials.

The releasing agents range from chlorhexidine (CHX) to silver diammine fluoride (SDF). CHX was mixed as a filler with resin monomers [71,72] for its broad-spectrum antibacterial capacity to prevent root caries biofilms formation (*Streptococcus mutans*, *Lactobacillus acidophilus*, *Actinomycetes*) [72,73]. However, CHX might disturb the oral micro-ecological balance [74] and its long-term antimicrobial activity relies on the recharge of CHX [72]. Ishiguro et al. [75] introduced SDF as a tooth surface coating agent and found that the SDF-coated root dentin exerted a strong inhibitory effect on the acid production by Streptococcus mutans through releasing silver, but the effect decreased after 1 week of aging. Hence, researchers turned their attention to non-releasing materials.

When the root lesions progress to cavities, carious tissue excavation and restorative treatment are required. As restorative materials used clinically do not have ideal antimicrobial properties, researchers attempt to incorporate antibacterial components into the adhesive systems [76,77,78] or restorative materials [79,80,81]. Quaternary ammonium methacrylates (QAMs) are a class of cationic compounds with a broad spectrum of antimicrobial activity [82,83], low capacity to induce drug resistance [84,85,86,87], and low toxicity [88]. The antimicrobial mechanism of QAMs was considered “contact killing”, since the positive quaternary amine charge could destroy the bacterial membrane which was negatively charged [89]. Compared with the releasing materials, QAMs can be copolymerized with the resin matrix [90] and fixed in the polymer network with a prolonged antimicrobial activity. Thomé et al. [91] found that the 12-methacryloyloxydodecylpyridinium bromide (MDPB)-containing composite inhibited the progression of artificial secondary root caries by Streptococcus mutans. Other novel multifunctional restorative materials have been developed in the laboratory by adding components with different capabilities. Zhou et al. [92] developed a nanocomposite with remineralizing and antibacterial properties via nanoparticles of amorphous calcium phosphate (NACP) and dimethylaminohexadecyl methacrylate (DMAHDM). The composite inhibited root caries biofilms of Streptococcus mutans [93,94], Lactobacillus acidophilus, and Candida albicans [95,96] in a recurrent root caries model and protected dentin hardness. These novel composite resins might successfully inhibit root caries in the elderly, control subgingival plaque, and reduce microleakage [97]. However, more comprehensive laboratory assessments, such as adhesive and anti-aging properties and further clinical evaluations are necessary before their widespread clinical applications.

### 4.2. Remineralization Materials

The elderly often suffer from gingival recession, causing the root surface to be exposed and more sensitive to acid attack. Since there is a decrease in the pH of the local oral microenvironment in this population, the teeth are more susceptible to demineralization. Generally, root caries in the elderly always develops rapidly and deeply, therefore, the remaining tooth structure is sometimes insufficient. As a result, restorative materials should have remineralizing properties. Due to the limited remineralizing properties of the glass ionomer cement and fluoride-releasing composite resin, various novel materials have been developed to promote the remineralization of demineralized enamel and dentin. Researchers have incorporated calcium phosphate compounds, fluoride, polyamidoamine (PAMAM), and bioactive glass (BAG) into glass ionomer and composite resin to improve their remineralizing properties. These novel materials could release calcium, phosphate, and fluoride ions to promote the remineralization of tooth hard structures. Since amorphous calcium phosphate (ACP) is the main component of hydroxyapatite, the development of restorative materials containing ACP has attracted widespread attention. The first to develop was the composite resin with ACP as an inorganic filler, which could release a large amount of calcium and phosphate ions. It had good hydrophilicity and biological safety. However, the mechanical properties of ACP were low, and the bonding properties of the material decreased significantly after water aging, resulting in the restoration fracture. Therefore, it cannot meet clinical needs for the elderly caries. With the emergence of nanotechnology, a variety of calcium phosphate nanoparticles as inorganic fillers [98,99], such as dicalcium phosphate anhydrous (DCPA), dicalcium phosphate dihydrate (DCPD), tetracalcium phosphate (TTCP), and amorphous calcium phosphate nanoparticles (NACP), have been gradually developed. The nano-amorphous calcium phosphate could meet the requirements for remineralization in the elderly. First of all, these agents were pH sensitive and released more calcium and phosphate ions in an acidic environment [100], thus responding to demineralization in a short time [101]. Moreover, they could recharge and supplement ions with calcium phosphate liquid to achieve long-term remineralization [102,103]. The mechanical properties of the nano material was still lower than the clinical universal composite resin material, not sufficient to satisfy the treatment of caries in the elderly. Nanocomposite containing CaF_2_ nanoparticles had pH sensitivity, released fluoride ions in a short time under low pH conditions, and effectively promoted remineralization. However, after 87 days, fluoride ion releasing was the same in different pH solutions, therefore, it lacked long-term pH-sensitive function [104]. The mechanical properties were better than those of the clinical resin-modified glass ionomer (RMGI) at the initial stage and after 2 years of water aging. The resin composite material containing the LiAl-F layered double hydroxide (LDH) had fluoride-release-and-recharge ability after 3 months of water aging. Moreover, its mechanical strength was better than RMGI [105]. Therefore, the above two materials could release a lot of fluoride ions and be recharged. However, the ability to release fluoride ions after long-term water aging, adhesive performance, etc., still required further experimental evidence. PAMAM dendrimers could be used as organic nucleation templates to induce biomimetic new-grown crystals on the demineralized dentin [106]. The novel composite resins containing NACP and PAMAM [107,108] could effectively promote the remineralization of root surface dentin. The BAG had a high ratio of calcium-to-phosphorus, promoting the formation of apatite crystals. Tezvergil-Mutluay et al. developed a fluoride-containing composite (BAG-F) [109]. BAG-F could induce the completely demineralized dentin surface to remineralize after 30 days. It could biologically inhibit MMP and the Cathepsin K (CTP), reducing the original protein crystals on the resin-tooth essence decomposition. Therefore, the composite resin containing PAMAM and BAG might meet the needs of caries remineralization in the elderly, but there was still a lack of long-term in vitro, animal, and clinical experiments to illustrate the remineralization function.

The above materials have been shown to have good remineralization ability. Some materials also have good mechanical properties, meeting the requirements of restorative materials for caries in the elderly. However, there is still a lack of long-term in vitro and clinical experiments to prove the remineralization ability, mechanical properties, and anti-aging properties of these novel materials.

### 4.3. Self-Healing and Low-Shrinkage Materials

The treatment of root caries in the elderly requires restorative materials with good antibacterial properties. The microleakage caused by the polymerization shrinkage and microcracks of the composite resin will increase plaque adhesion, causing secondary caries. Therefore, reducing the polymerization shrinkage and timely repair of the microcracks are potential strategies. Novel materials have been developed to deal with these two issues, including self-healing composite resins and low polymerization shrinkage composite resins.

The self-healing composite resin has self-healing microcapsules. When the resin cracks, the microcapsules are broken to release the healing fluid to repair the cracks. Wu et al. developed a self-healing composite resin using triethylene glycol dimethacrylate (TEGDMA)-*N*,*N*-dihydroxyethyl-p-toluidine (DHEPT) (TEGDMA-DHEPT) as the healing fluid. When the microcracks generated, benzoyl peroxide (BPO) in the resin matrix triggered TEGDMA to repair the cracks. George Huyang has developed a new type of SHDC [110]. The microcapsules contained a healing powder (HP, strontium fluoroaluminosilicate particles) and a healing fluid (HL, polyacrylic acid aqueous solution). As the microcracks developed, they would release HL. This liquid then reacted with the HP particles exposed through the crack formation process to form an insoluble reaction product filling and sealing the crack. The self-healing resin had a self-healing rate of up to about 90%, and could repair most cracks. Moreover, the incorporation of microcapsules did not affect the mechanical properties of the material. Therefore, it might be a method to solve the microcracks in composite resins, especially in the elderly.

Concerning the polymerization shrinkage of composite resins, low polymerization shrinkage composite resins have been developed. Young Park et al. developed the norbornene sulfide (MDNS)-phthalate allyl sulfide (PAS) material, incorporating allyl sulfide functional groups into the norbornene-methacrylate comonomer resin. The presence of allyl sulfide allowed the addition-fragmentation chain transfer (AFCT) to reduce the stress in methacrylate-based systems (stress reduction of more than 96%), while retaining excellent mechanical properties. Wang et al. incorporated methacrylic polyhedral oligomeric silsesquioxane (POSS) into a new type of nano-SiO2 dental composite resin. The polymerization shrinkage volume of the conventional composite resin was about 1.5 times that of POSS. POSS could also strengthen the elastic modulus and hardness of the composite resin [111]. Concerning caries in the elderly, low-polymerization shrinkage materials could reduce polymerization shrinkage, thereby reducing the breakage and microleakage of the restoration [112]. However, due to the complex and ever-changing internal environment of the oral cavity, in vivo and clinical experiments are still required.

In summary, many novel restorative materials have been developed. Some have already been used in clinics, such as fluoride-releasing composites and short fiber-reinforced composites. Fluoride-releasing composites might provide antibacterial and remineralizing properties, but further research is needed. Short fiber-reinforced composites increase toughness and other physical properties of the material compared to conventional composite resins [113]. However, these materials are not enough to meet all the requirements of root caries restoration in the elderly. Other novel materials which have not been used in clinical practice have antibacterial properties, some have remineralization properties, and some can repair microleakage and reduce polymerization shrinkage. For the caries of the elderly, these novel restorative materials can improve the deficiencies of the commonly used restorative materials in clinics in one or more aspects, but unfortunately there is no one specific for the caries of the elderly. Therefore, further research focusing on the restorative materials for caries in the elderly is necessary.

## 5. Conclusions

With the evolution of dental caries treatment to accommodate personalized treatments, patients have increasing expectations on the therapeutic effect of restorative materials for specific caries. Novel restorative materials with antimicrobial, remineralizing, and other properties were designed and synthesized. However, few studies have evaluated the restorative materials of caries in the elderly, especially root caries. In addition, the intelligent control of the release of antimicrobial materials has received little attention. Furthermore, the problems of moisture isolation, microleakage, and aging resistance of restorative materials have not been satisfactorily solved. Therefore, the restorative materials specifically for caries in elderly populations need to be studied and then applied in clinics, which is expected to offer tremendous benefits to geriatric oral health.

## Figures and Tables

**Table 1:** Comparison of currently available restorative materials for elderly populations.

	Antibacterial Property	Adhesive Property	Remineralization Property	Mechanical Property	Anti-Aging Property	Aesthetic Property
Amalgam	+	−	−	+	+	−
Conventional Glass Ionomer Cement	+	+	+	−	−	−
High-Viscosity Glass Ionomer Cement (Ketac Molar Easymix, etc.)	+	+	+	+	−	−
Resin-modified Glass Ionomer Cement (Fuji II LC, etc.)	+	+	+	−	+	+
Conventional Light Curing Composite Resin	−	− (Without adhesive system)	−	+	−	+
Fluoride-releasing composite (Compomer, e.g., Dyract Extra, etc.; Giomer, e.g., Beautifil II, etc.)	Further studies are needed	− (Without adhesive system)	Further studies are needed	+	−	+
Short Fiber-reinforc ed composite (everX Flow, everX Posterior, etc.)	Further studies are needed	− (Without adhesive system)	Further studies are needed	+	+	+

“+” Indicates that the property is clinically acceptable; “−” indicates the opposite.
